# Biochemical analysis of antimicrobial peptides in two different *Capsicum* genotypes after fruit infection by *Colletotrichum gloeosporioides*

**DOI:** 10.1042/BSR20181889

**Published:** 2019-04-23

**Authors:** Álan C. Maracahipes, Gabriel B. Taveira, Erica O. Mello, André O. Carvalho, Rosana Rodrigues, Jonas Perales, André Teixeira-Ferreira, Marciele S. Silva, Gustavo L. Rocha, Kátia Valevski Sales Fernandes, Valdirene M. Gomes

**Affiliations:** 1 Laboratório de Fisiologia e Bioquímica de Microrganismos, Centro de Biociências e Biotecnologia, Universidade Estadual do Norte Fluminense Darcy Ribeiro, Campos dos Goytacazes, RJ, Brazil; 2 Laboratório de Melhoramento e Genética Vegetal, Centro de Ciências e Tecnologias Agropecuárias, Universidade Estadual do Norte Fluminense Darcy Ribeiro, Campos dos Goytacazes, RJ, Brazil; 3 Laboratório de Toxinologia, Fundação Osvaldo Cruz (FIOCRUZ), Rio de Janeiro, RJ, Brazil; 4 Laboratório de Química e Função de Proteínas e Peptídeos, Universidade Estadual do Norte Fluminense Darcy Ribeiro, Campos dos Goytacazes, RJ, Brazil

**Keywords:** anthracnose, Defensin, Pepper, PR proteins, trypsin inhibitors

## Abstract

There are several phytosanitary problems that have been causing serious damage to the *Capsicum* crops, including anthracnose. Upon attack by certain pathogens, various protein molecules are produced, which are known as proteins related to pathogenesis (PR proteins), including antimicrobial peptides such as protease inhibitors, defensins and lipid transfer proteins (LTPs). The objective of this work is to identify antimicrobial proteins and/or peptides of two genotypes from *Capsicum annuum* fruits infected with *Colletotrichum gloeosporioides*. The fungus was inoculated into *Capsicum* fruits by the deposition of a spore suspension (10^6^ conidia ml^−1^), and after 24 and 48 h intervals, the fruits were removed from the humid chamber and subjected to a protein extraction process. Protein analysis of the extracts was performed by tricine gel electrophoresis and Western blotting. The distinctive bands between genotypes in the electrophoresis profiles were subjected to mass spectrometry sequencing. Trypsin inhibition assays, reverse zymographic detection of protease inhibition and β-1,3-glucanase activity assays were also performed and extracts were also tested for their ability to inhibit the growth of *C. gloeosporioides* fungi ‘*in vitro’*. There were several low molecular weight proteins in all treated samples, and some treatments in which antimicrobial peptides such as defensin, lipid transfer protein (LTP) and protease inhibitor have been identified. It was shown that the green fruits are more responsive to infection, showing the production of antimicrobial peptides in response to injury and inoculation of the fungus, what did not occur in ripe fruits under any treatment.

## Introduction

Sweet and chili peppers belong to the Solanaceae family and to the *Capsicum* genus, which encompasses more than 31 described species, with only five being domesticated (*Capsicum annuum* var. *annuum, C. baccatum* var. *pendulum* and *umbilicatum, C. chinense, C. frutescens* and *C. pubescens*) [1]. During the cultivation of *Capsicum*, the plants may be vulnerable to several pests and diseases, what may lead to severe losses for farmers. Among these diseases is the anthracnose.

Anthracnose is a disease of complex etiology, caused by distinct isolates of different species of *Colletotrichum* [2]. It is one of the most destructive diseases of sweet and chili peppers in tropical and subtropical regions, and its spread occurs mainly in crops that are grown outdoors during periods of high temperature and humidity. The fungus produces conidia (spores) as reproductive structures, which are released and disseminated by splashes of rain, wind, and insects, among other vectors [3].

In a favorable environment for their development, the conidia germinate, producing the appressorium at the end of the germ tube or at the end of the hyphae of the mycelium, which are differentiated structures used to force entry into the surface of the plant. Thus, the fungus penetration into the host tissue, what follows with tissue colonization with rapid hyphae growth [4]. The disease mainly affects the fruits, causing necrotic lesions of circular shape, with dark coloration and variable diameters, from which a mucilaginous mass of orange color evolves, i.e. the conidia [2].

Several plant proteins are produced upon pathogens attack, some of which have low molecular masses, below 10 kDa, and a broad spectrum of action [5]. These molecules are known as proteins related to pathogenesis or PR proteins, which may be particularly enriched in certain families, such as protease inhibitors (PR-6), β-1,3-glucanase (PR-2), defensins (PR-12), thionins (PR-13), and lipid transfer proteins (LTPs) (PR-14). Among these families are also the antimicrobial peptides (AMPs), which have been especially prominent in recent years, gaining attention in researches related to plant defenses [6]. Several of these peptide families may also be present at constitutive levels in various plant tissues, especially in seeds, thus contributing to the resistance of healthy plants against various pathogens. These AMPs can be expressed in high concentrations upon the aggression of some pathogen or upon exposure to biotic or abiotic stresses. Effectively, antimicrobial peptides tend to act directly in the region of injury against the aggressor and can be found in several plant organs, such as seeds, reproductive organs, tubers, fruits and flowers, and in almost all plant species [7].

AMPs may have activity against various bacteria, fungi, viruses, and parasites and represent rapid and effective defense establishment capability. The storage of AMPs in plants can play a particularly essential role in protecting young plants that are vulnerable during the early stages of their life cycle [8]. It is evident that antimicrobial peptides are an important tool in the defense of plants against several injuries and that they present great potential for use in the launching of resistant cultivars, but more studies are necessary for a better comprehension of their mechanism of action to facilitate their *in vivo* use in agronomic applications. Thus, the objective of this work was to identify proteins and antimicrobial peptides in fruits of two different genotypes of *Capsicum annuum* infected with *Colletotrichum gloeosporioides* fungus.

## Materials and methods

### Plants and fruit picking

UENF 1381 and ‘Ikeda’ had been evaluated for disease resistance, including bacterial spot (*Xanthomonas* spp.) and anthracnose (*Colletotrichum gloeosporioides*). UENF 1381 is a *Capsicum annuum* var *annuum* accession from the UENF genebank, it is pungent and identified as resistant to bacterial spot [9] and anthracnose. ‘Ikeda’ is a very traditional sweet pepper (*Capsicum annuum*) genotype that has been used by small farmers for a long time, and it is susceptible to bacterial spot and also to anthracnose [10].

The experiment was carried out from June 2015 to December 2016 at the *Universidade Estadual do Norte Fluminense Darcy Ribeiro* (UENF), located in the Campos dos Goytacazes municipality, Rio de Janeiro, Brazil. Seeds of *C. annuum* from the UENF1381 accession and the cultivar Ikeda, from the Genebank of the *Laboratório de Melhoramento Genético Vegetal (LMGV)*, were seeded in polystyrene foam of 128 cells containing commercial substrate and maintained in a growth chamber with a controlled temperature of 28°C and a photoperiod of 12 h. After the seedlings exceeded 10 cm in height, they were transplanted into 5 L pots and placed in a greenhouse.

The flowers were marked during the anthesis, after a period of 30 days for immature fruits and 45 days for ripe fruits, the marked fruits were collected and taken to the *Laboratório de Fisiologia e Bioquímica de Microrganismos (LFBM)* for disinfestation, inoculation and extraction of the proteins from fruits. UENF1381 access was selected as a resistant genotype, and Ikeda cultivar was selected as susceptible genotype (Table 1).

**Table 1:** Samples derived from control (not inoculated) or inoculated fruits with the fungus *Colletotrichum gloeosporioides*, at intervals of 24 and 48 h after inoculation (HAI)

Genotypes	Feature	Samples	Identification
UENF138	Resistant to anthracnose and bacterial spot	Inoculated Immature fruit (24 HAI)	IIF24
		Control immature fruit (24)	CIF24
		Inoculated Immature fruit (48 HAI)	IIF48
		Control immature fruit (48)	CIF48
		Inoculated ripe frit (24 HAI)	IRF24
		Control ripe fruit (24)	CRF24
		Inoculated ripe fruit (48 HAI)	IRF24
		Conrol ripe fruit (48)	CRF48
IKEDA	Susceptible to anthracnose, *Pepper yellow mosaic virus*, and bacterial spot	Inoculated immature fruit (24 HAI)	IIF24
		Control immature fruit (24)	CIF24
		Inoculated Immature fruit (48 HAI)	IIF48
		Control immature fruit (84)	CIF48
		Inoculated ripe fruit (24 HAI)	IRF24
		Conrol ripe fruit (24)	CRF24
		Inoculated ripe frit (48 HAI)	IRF48
		Conrol ripe fruit (48)	CRF48

### Obtaining conidia solution of *Colletotrichum gloeosporioides*

The isolate of *C. gloeosporioides* was given in by LMGV-UENF, cultured in Petri dishes containing Potato Dextrose Agar (PDA), and maintained in an incubator at a temperature of 28 ± 2°C for 7 days at 28°C with a 12-h photoperiod. For inoculation of the fungus into *C. annuum* fruits, a spore solution was made and adjusted using a Neubauer chamber at a concentration of 10^6^ conidia ml^−1^ [11].

### Inoculation of *Colletotrichum gloeosporioides* into fruits

Immature and ripe fruits ranging from 2 to 5 cm in length and 1 to 1.5 g per fruit were disinfected in 70% alcohol, 0.5% sodium hypochlorite and ultrapure water, for 1 min at each stage. An injury was created in the middle region of each fruit with a sterilized needle and this point was used for the inoculation with 20 μl of the *C. gloeosporioides* fungus spore solution. Control (not inoculated) fruits were established, in which a drop of ultrapure water was deposited after injury. The trays with the fruits were packed in a transparent plastic box with a lid 28°C containing cotton wads wetted with water to form a humid chamber [1] (Supplementary Figure S1). Forty fruits were inoculated in each case, and all these processes were done in triplicate.

### Extraction fruit proteins

Protein extraction was performed according to the methodology proposed by Taveira et al. [12], in which immature and ripe fruits were removed from the humid chamber at two different intervals: 24 and 48 h after inoculation (Table 1). The peduncle and seeds of all fruits were removed and discarded.

About 40 g of inoculated fruits (without peduncle and seeds) and 40 g of non-inoculated fruits (without peduncle and seeds) (controls) from each interval were used for protein extraction The fruit powders were processed in 200 ml of extraction buffer (10 mM Na_2_HPO_4_, 15 mM NaH_2_PO_4_, 100 mM KCl, 1.5% EDTA, pH 5.4) (Sigma) for 15 min with a multiprocessor, and the mixture was shaked for 2 h at 4°C. The suspension was centrifuged at 15,400 × ***g*** for 45 min at 4°C and then the supernatant was filtered with a filter paper; the pellet was discarded. Ammonium sulfate at 70% saturation was added to the solution, which was shaken for 40 min and kept at 4°C overnight. The next day, the solution was centrifuged at 15,400 × ***g*** for 45 min at 4°C and the supernatant was discarded. The precipitated material was resuspended and placed in a water bath for 15 min at 80°C before centrifuged at 15,400 × ***g*** for 30 min. The final precipitate was discarded and the supernatant was dialyzed using semipermeable membranes (exclusion cutoff 1000 Da) at 4°C for a period of 3 days using distilled water, with three exchanges of water daily. After this period, the obtained samples were lyophilized, resuspended in water, conditioned in microcentrifuge tubes and stored at −18°C. Quantitative protein determinations were done by the Bradford method [13] with bovine serum albumin as the standard.

### Tricine gel electrophoresis

The 1D gel was assembled according to the methodology proposed by Schagger and Von Jagon [14], where 20 μg ml^−1^ of sample from each treatment was prepared with 5% sample buffer and β-mercaptoethanol (1%), totaling 20 μl. The samples were placed in a water bath at 80°C for 5 min, centrifuged at 15,000 × ***g*** for 3 min and then applied to each well of the gel. The run was performed under an amperage of 400 A and voltage between 22 and 26 V overnight. At the end of the run, the gel was removed from the glass plates and immersed in Coomassie R dye solution (0.05% Coomassie-brilliant blue R, 40% methanol, 7% acetic acid), then in a destaining solution (40% methanol, 7% acetic acid); the gel image was recorded with the aid of a gel documentation system.

### Western blotting

After the tricine gel run, the gels were withdrawn from the plates and immersed in transfer buffer (182 mM glycine, 25 mM Tris and 20% methanol) for 20 min. Similarly, nitrocellulose membranes (cut to the same size as the gels) were immersed in transfer buffer for 20 min. Proteins from the gels were electroblotted onto nitrocellulose membranes using a commercial (semi-dry) transfer cell. A ‘sandwich’ was mounted consisting of the filter paper, membrane and tricine gel in the transfer tank, in the following order: five filter papers, membrane, gel, five filter papers. The transfer run lasted for 2 h at maximum voltage and 56 VA.

After transfer, the ‘sandwiches’ were carefully disassembled, and the membranes were reversibly stained with Ponceau S (0.1%) to determine the success of the transfer. The membranes were then subjected to the following treatments: immersion in blocking buffer (10 mM NaH_2_PO_4_ (PBS pH 7.4) + 0.15 M NaCl + 2% skim powdered milk) at 30°C for a period of 1 h, with the aim of blocking non-specific sites on the membrane; incubation in blocking buffer containing the primary antibody polyclonal against the purified LTP from *C. annuum* seeds [15] (anti-LTP at a titer of 1:1000) for a period of 16 h at 4°C; washing in PBS pH 7.4 at 30°C (five washes of 10 min each); incubation in blocking buffer, this time containing the secondary antibody (rabbit anti-IgG conjugated to peroxide, 1:1000), for a period of 2 h at 30°C; and washing in PBS pH 7.4 at 30°C (five washes of 10 min each). The immune reactions on the membrane were developed by immersing the membranes in a 10 mg ml^−1^ solution of 3,3′-diaminobenzidine (DAB) with 10 μl of hydrogen peroxide [16].

### Determination of β-1,3-glucanase activity

The copper reagent for the determination of reducing sugars was prepared according to a method described by Somogy [17], and the arsenomolybdate reagent was prepared according to a method described by Nelson [18]. The determination of β-1,3-glucanase activity in the samples was done according to a method described by Fink et al. [19]. The reagents were added in test tubes containing 20 µg ml^−1^ of proteins, 125 μl of laminarin (2 mg ml^−1^ in 50 mM sodium acetate buffer, pH 5.0) and adjusted to a final volume of 500 μl with 50 mM sodium acetate buffer, pH 5.0. The mixture was incubated at 37°C in an incubator for 12 h. After the incubation period, 500 ml of the copper reagent was added, and the mixture was boiled for 10 min, then placed at room temperature, and 1000 μl of the arsenomolybdate reagent was added. For a reaction control, the protein sample was replaced by assay buffer. A unit of glucanase activity was defined as the concentration of the enzyme that yields an absorbance of 0.001 when read at 500 nm.

### Trypsin inhibition assay

The trypsin inhibitory activity of the UENF1381 and IKEDA samples (Table 1) was measured based on the hydrolytic activity of bovine commercial trypsin over N-benzyol-d,l-arginine p-nitroanilide (BAPNA) substrate after incubation with the extracts. Tris-HCl buffer (50 mM, pH 8.0), 25 μl of BAPNA substrate (5 mM), 20 μg of samples and 10 μl of trypsin (1 µg ml^−1^) were placed in microcentrifuge tubes. There were alternative blank treatments for all samples tested, without the addition of either the substrate or the enzyme. The tubes were incubated in a water bath at 37°C for 30 min. To stop the reaction, 100 μl of acetic acid was added. Afterward, photometric reading of the treatments was performed on the basis of released *p*-nitroanilide at wavelength of 405 nm [20].

### Reverse zymographic detection of protease inhibition

Inhibition tests of trypsin on gel were performed using a methodology of Felicioli et al. [21], where the samples were separated on polyacrylamide gel (12% SDS/PAGE) co-polymerized with 0.1% gelatin, under semi-denaturing conditions (sample buffer did not contain SDS, urea or β-mercaptoethanol). After an electrophoretic run, the gels were placed in wash buffer (0.1 M Tris-HCl, pH 8.0 containing 2.5% Triton X-100) twice for 60 min to remove SDS. They were immersed in incubation buffer (50 mM Tris-HCl, pH 8.0 containing 20 mM CaCl_2_ and 50 µg ml^−1^ trypsin) at 37°C for 1 h. The gels were then rinsed with distilled water for removal of the trypsin excess. The non-digested proteins were stained by a solution of 0.2% Coomassie Brilliant Blue G 250, 45% methanol and 10% acetic acid for 30 min, and destained thereafter. The dark blue bands on the gel show the potential presence of protease inhibitors, indicating the inability of trypsin to digest gelatin in those regions.

### Effect of the extracts on fungal growth

The fungus *C. gloeosporioides* was transferred from stock to a Petri dish containing PDA medium and grown for approximately 7 days at 28°C. After this period, 10 ml of PDA media were poured over the plate containing the fungus, and the conidia were released with the aid of a Drigalski spatula. This suspension was filtered through gauze to prevent the passage of mycelial debris. These conidia were quantified in a Neubauer chamber (Laboroptik) under an optical microscope.

A quantitative assay for fungal growth inhibition was performed following the protocol developed by Broekaert et al. [22] with modifications, as follows. To verify the effect of protein extractions on *C. gloeosporioides* growth, 1 × 10^4^ conidia ml^−1^ in 200 µl of PDA medium were incubated at 25°C in 96-well microplates (Nunc) in the presence of protein extracts at a concentration of 50, 100 and 200 µg ml^−1^. Optical readings at 620 nm were collected at the start and at every 6 h for 60 h. Fungal growth in medium without the addition of protein extracts was determined as control. Graphics of absorbance versus time were plotted. The experiments were done in triplicate and repeated three times, and the inhibition percentage was calculated by the formula according to Vieira et al*.* [23] with the following modifications: the inhibition percentages were assessed against a control representing 100% growth based on the formula [100 – (ABS620 × 100/ cABS620)], where ABS620 was the average absorbance reading at 620 nm of protein extraction-treated cells at 60 h, and cABS620 was the average absorbance reading at 620 nm of the control cells at 60 h.

### Amino acid sequencing by mass spectrometry analysis

For internal sequencing of the *Capsicum* fruits peptides, after electrophoresis in tricine gel, the gel was removed and subjected to staining and destaining steps. Protein bands of interest were extracted and digest from the gel and subjected to a mass spectrometry evaluation [24]. In brief, the peptides digest by trypsin was first co-crystalized with a large molar excess of the α-cyano-4-hydroxycinnamic acid matrix before being analyzed by matrix-assisted laser desorption time-of-flight mass spectrometry (MALDI-TOF-MS), using an AB SCIEX TOF/TOFTM 5800 System spectrometer (AB SCIEX) in the reflection mode. Up to 10 of the most intense ion signals with a signal-to-noise ratio above 20 were selected as the precursors for MS/MS. External calibration in MS mode was performed using a mixture of four peptides: des-Arg1-Bradykinin (*m/z* = 904.47), angiotensin I (*m/z* = 1296.69), Glu1-fibrinopeptide B (*m/z* = 1570.68) and ACTH (18–39) (*m/z* = 2465.20). MS/MS spectra were externally calibrated using known fragment ion masses observed in the MS/MS spectrum of Glu1-fibrinopeptide B. MS/MS database searching was performed against the NCBIprot databases using the Mascot software (www.matrixscience.com). The search parameters included two missed tryptic cleavages allowed and non-fixed modifications of methionine (oxidation) and fixed cysteine (carbamidomethylation). Searches for sequence homology were performed with the BLAST program [25].

### Statistical analysis

Data from trypsin assays, glucanase assays and fungal growth inhibition were evaluated using one-way ANOVA. Mean differences at *P* < 0.05 were considered to be significant. All statistical analyses were performed using the Graph Pad Prism software (version 5.0 for Windows). The IC_50_ was calculated based on a linear regression curve, and it was defined as the protein extracts concentration required to inhibit 50% of microorganism growth in the conditions tested.

## Results and discussion

The fungus *C. gloeosporioides* is the main responsible for anthracnose in fruits of *Capsicum* in Brazil, and can appear in any part of the plant, from its development in the field to even in fruits stored in the post-harvest phase [26]. *Colletotrichum* species can infect pepper fruits at any stage of maturation. There are studies showing a strong incidence of *C. acutatum* and *C. gloeosporioides* in immature fruits [27,28]. Kim et al. [29] found several accessions of *Capsicum* resistant to *C. acutatum*, but only in immature stage of the fruits. In order to detect different proteins and peptides, which could be differentially expressed in ripe and immature fruits, two distinct genotypes of *Capsicum* with different resistance levels toward the fungus were chosen.

**Figure 1:**
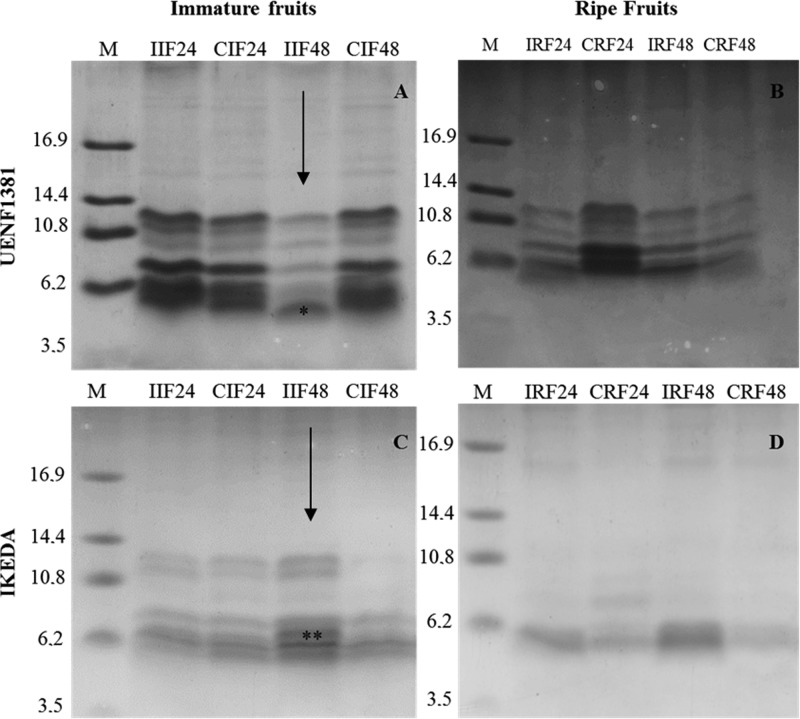
Tricine gel electrophoresis from UENF1381 and the IKEDA cultivars Tricine gel electrophoresis showing low molecular weight protein pattern of *Capsicum annuum* extracts from UENF1381 and the IKEDA cultivars in response to the fungus *Colletotrichum gloeosporioides*. M: low molecular weight marker; I: inoculated; C: control (not inoculated); 24: 24 h after inoculation; 48: 48 h after inoculation; *Defensin; ** Protease inhibitor.

The pattern of the low molecular weight proteins of the ripe and immature fruits of *C. annuum* inoculated with *C. gloeosporioides*, at 24 and 48 h after inoculation, was analyzed by tricine gel electrophoresis (Figure 1). In the fruits of UENF1381, all the treatments showed a similar protein profile, with at least five bands ranging from 6 to 12 kDa, except for the IIF48 treatment, which presented an additional band of 5 kDa. This band was subjected to mass spectrometry sequencing. For the Ikeda genotype, the immature fruits showed a different protein profile compared with the ripe fruits, where the immature fruits under different treatments showed at least five bands of 5 to 12 kDa, while the ripe fruits presented bands of 5 to 10 kDa. The IIF48 treatment showed a band at approximately 7 kDa, which was also subjected to mass spectrometry sequencing.

**Figure 2:**
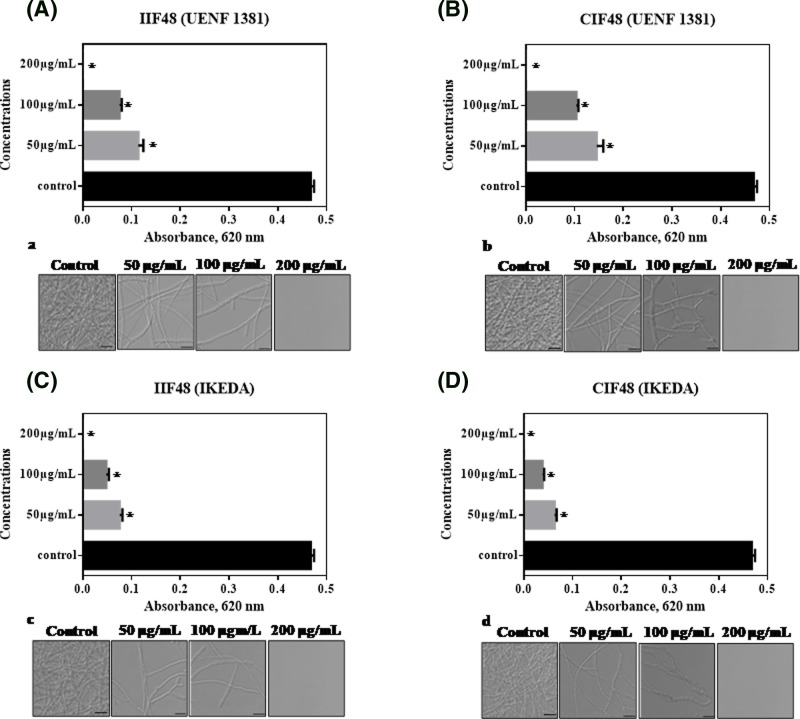
The effect of protein extracts of UENF1381 and IKEDA on the growth of plant pathogen *Colletotrichum gloeosporioides* Protein extracts IIF48 (**A**) and CIF48 (**B**) of UENF1381, and IIF48 (**C**) and CIF48 (**D**) of IKEDA at concentrations of 50, 100 and 200 µg ml^−1^. (*) indicates significant differences by the one-way Tukey test (*P* < 0.05). Images of *C. gloeosporioides* cells by light microscopy after different incubation times with IIF48 (a) and CIF48 (b) of UENF1381 and IIF48 (c) and CIF48 (d) of IKEDA. Control cells without protein extractions. Bars = 20 μm, 63× objective. Experiments were performed in triplicate.

*Capsicum* extracts have been shown to inhibit the growth and hyphae formation of various phytopathogenic fungi. In the inhibition test of the filamentous fungus *C. gloeosporioides* (Figure 2), different extracts IIF48 (A) and CIF48 (B) of UENF1381, and IIF48 (C) and CIF48 (D) of Ikeda were tested at different concentrations (50, 100 and 200 μg ml^−1^). The fungal growth was inhibited by all extracts and at all concentrations tested. From 50 μg ml^−1^, *C. gloeosporioides* growth inhibition was observed in all the extracts tested, with values ranging from 75.64% (IIF48) to 100% inhibition when the concentration used was 200 μg ml^−1^. The fractions IIF48 and CIF48 from Ikeda showed a higher percentage of inhibition at all tested concentrations when compared with fractions IIF48 and CIF48 from UENF1381. The fungal growth inhibition tests were corroborated by the images obtained by optical microscopy (Figure 2a–d), in which fewer *C. gloeosporioides* hyphae were observed. At 200 μg ml^−1^ extracts, total absence of hyphae was perceived. Taveira et al. [30] isolated an antimicrobial peptide of *Capsicum annuum*, belonging to the thionin family of peptides, called *CaThi*, which inhibited 83% of the growth of *Fusarium solani* at the concentration of 50 μg ml^−1^. Additionally, the authors showed *CaThi* prevents hyphae formation and permeates membranes of the pathogen. The results about antimicrobial activity reaffirm the richness of antimicrobial peptides in *Capsicum* extracts, and their potential for use against various phytopathogenic fungi of agronomic interest.

The sequencing of the 5 kDa band in the IIF48 treatment (highlighted by * in Figure 1) from fruits of UENF1381 was performed by mass spectrometry, and a sequence fragment with 40 amino acid residues was obtained, later named *Ca*Def1. Alignment of the fragment called *Ca*Def1 (Table 2) showed that it had 90–100% similarity to the above-described defensin sequences. Similar antimicrobial peptides by alignment were flower-specific defensin-like *Capsicum annuum* (GenBank XP_016579688.1), defensin-like protein *C. chinense* (NCBI Reference Sequence: XP_016579688.1), Defensin-like protein *C. baccatum* (NCBI Reference Sequence: PHT42128.1) and flower-specific defensin-like *C. baccatum* (NCBI Reference Sequence: XP_016579687.1.). It can be seen that all six cysteine residues (Cys) are well conserved in all aligned sequences. Van der Weerden et al. [31] performed the alignment of 139 plant defensins, separated into 17 groups, in which it was possible to visualize the conserved amino acid regions inside and outside the groups, mainly the positions of the eight Cys residues of all the peptides, which were completely conserved. In a study done by Rogozhin et al. [32], where the objective was to isolate and characterize seed defensins from the Ranunculaceae family, two sequences with 30 amino acids were obtained, containing eight residues of Cys. On the basis of the alignment, it was concluded that these sequences were defensins, named Ns-D1 and Ns-D2. An inhibition assay was performed, and Ns-D1 and Ns-D2 showed high inhibition activity against the hyphae of *Bipolaris sorokiniana, Fusarium solani* and *Botrytis cinerea*, as well as cause rupture in spores of *B. sorokiniana* that had already germinated, after 48 h of incubation.

**Table 2:** Alignment of amino acid residues from immature fruits of the UENF1381

Identification	Sequences	%*I*
*CaDef1*	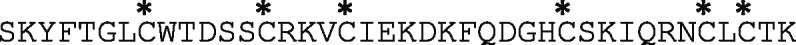	–
Flower-specific defensin-like *C. annuum*	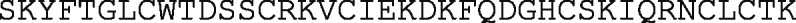	100%
Defensin-like *C. chinense*	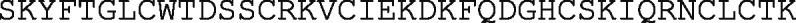	98%
Defensin-like *C. baccatum*	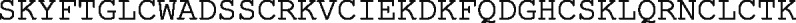	95%
Flower-specific defensin-like *C. baccatum*	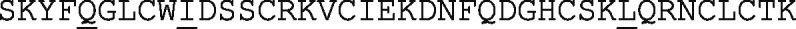	90%

Alignment of 40 amino acid residues from the 5 kDa peptide of immature fruits of the UENF1381 cultivar of *Capsicum annuum*, 48 h after inoculation with the fungus *Colletotrichum gloeosporioides*. The Cys residues are conserved in all aligned sequences, being highlighted by *. The different amino acid residues between aligned sequences are underlined. The sequences were obtained from SWISS-PROT and aligned by Clustal Omega. The 5 kDa peptide was named *Ca*Def1 (*Capsicum annuum* Defensin 1) and showed homology with the sequences: Flower-specific defensin-like *Capsicum annuum*, GenBank XP_016579688.1; Defensin-like protein *C. chinense*, NCBI Reference Sequence: XP_016579688.1; Defensin-like protein *C. baccatum*, NCBI Reference Sequence: PHT42128.1; Flower-specific defensin-like *C. baccatum*, NCBI Reference Sequence: XP_016579687.1.

Carvalho and Gomes [33] showed that defensins are common in the plant kingdom and are involved in several *in vitro* activities, acting as microbial inhibitors, protease inhibitors, α-amylase inhibitors, translation process inhibitors, zinc tolerance mediators, enzymes and even as ion channel blockers. These studies show the great potential of defensins as defense proteins in plants against several pathogens, stressing their importance and the need for studies for its further application in agronomic scenarios.

A ±7 kDa band of the IKEDA fruit treatment IIF48 (highlighted by ** in Figure 1) was also subjected to mass spectrometry sequencing, and a 16 amino acid residue fragment was obtained, which, after alignment, showed similarity to serine protease inhibitors (Table 3). This peptide was named *Ca*TI2 because of its homology to a pepper seed peptide previously isolated and determined to be a trypsin inhibitor, called *CaTI* [34]. The fragment was aligned with the following proteins: protease inhibitor IB-like of *C. annuum* (NCBI reference sequence: XM_016687591), trypsin inhibitor 1-like of *C. annuum* (NCBI reference sequence: XM_016689257.1), trypsin inhibitor 1-like of *Nicotiana sylvestris* (NCBI reference sequence: XM_009776647.1), and trypsin inhibitor 1-like of *Solanum tuberosum* (NCBI reference sequence: XM_006363611.1). In a work done by Mishra et al. [35], *C. annuum* leaves were subjected to various treatments, such as aphid infestation or mechanical injury with added oral secretion (OS) of *Helicoverpa armigera* or water. Silva et al. [8] showed that a trypsin inhibitor isolated from *C. annuum* seeds showed inhibitory activity against phytopathogenic fungi such as *F. oxysporum, C. gloeosporioides* and *C. lindemuthianum*, hindering their growth and causing morphological changes in the hyphae, reaffirming the important role of protease inhibitors in plant defense and as a possible tool in plant breeding for pest and disease resistance.

**Table 3:** Alignment of amino acid residues from immature fruits of the IKEDA

Identification	Sequences	%*I*
CaIkdTi	KDWWPELLGVPAGLAR	–
Proteinase inhibitor I-B-like	KDKWPELLGVPAGLAR	94%
Trypsin inhibitor 1-like Ca	KDTWPELLGVPAKLAR	94%
Trypsin inhibitor 1-like Ns	KDTWPELLGVPAKLAR	88%

Alignment of 16 amino acid residues from the 7 kDa peptide of immature fruits of the IKEDA cultivar of *Capsicum annuum*, 48 h after inoculation with the fungus *Colletotrichum gloeosporioides*. The sequences were obtained from SWISS-PROT and aligned by Clustal Omega. The 7 kDa peptide was named *Ca*TI2 (*Capsicum annuum* trypsin inhibitor). The different amino acid residues between aligned sequences are underlined. The I-B-like protease inhibitor of *Capsicum annuum*, NCBI Reference Sequence: XM_016687591.1; trypsin inhibitor 1-like Ca of *Capsicum annuum*; NCBI Reference Sequence: XM_016689257.1; Trypsin inhibitor 1-like Ns of *Nicotiana sylvestris*, NCBI Reference Sequence: XM_009776647.1; Trypsin inhibitor 1-like St of *Solanum tuberosum*, NCBI Reference Sequence: XM_006363611.1.

**Figure 3:**
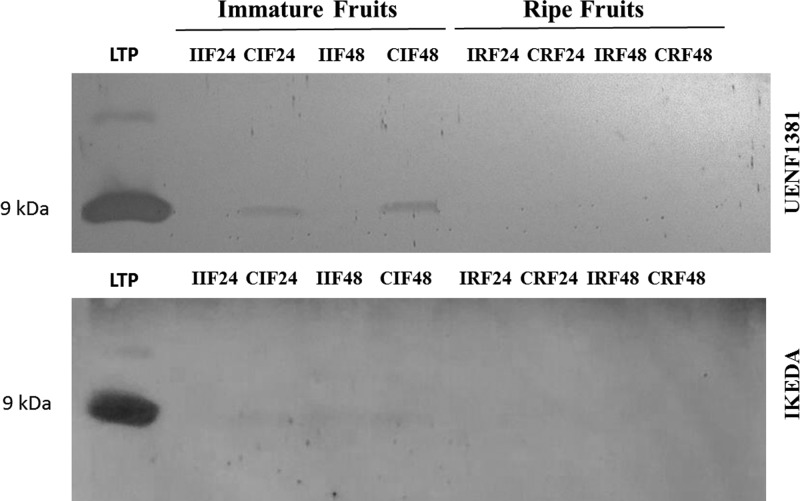
Detection of LTPs in immature and ripe fruits of *Capsicum annuum* Western blotting for the detection of LTPs in immature and ripe fruits of *Capsicum annuum* (UENF1381 and IKEDA) from the UENF BAG, inoculated with fungus *Colletotrichum gloeosporioides*, at 24 and 48 h after inoculation. Membrane revealed with DAB. LTP: Control with purified LTP of seeds of *Capsicum annuum*; I: inoculated; C: Control (not inoculated).

Analyzing Western blotting using anti-LTP antibody and a pure 9 kDa LTP of *Capsicum* seeds as control, it was possible to visualize two bands of the same size as the control in the treatments CIF24 and CIF48 (Figure 3). Our results show that these bands reacted positively with the anti-LTP antibody, suggesting that the protein in the band is immunorelated to LTPs. It is observed that in these immature fruits, inoculation with the fungus *C. gloeosporioides* is associated with a decrease in the expression of this protein, but we cannot affirm that LTP promoted or decreased resistance in this case. In the Ikeda (susceptive genotype) fruits, there was a poor positive reaction in treatments of CIF24, IIF48 and CIF48, and there were no positive reactions with the anti-LTP antibody in the ripe fruit treatments. Diz et al. [36] identified an LTP in *C. annuum* seeds called *Ca-LTP1*, which has inhibitory activity and can cause several morphological changes in the fungus *C. lindemunthianum*, an anthracnose fungus found in several cultivated species, for example, in *Phaseolus vulgaris* L., and may cause up to 100% losses in the production and in the vegetative parts of the plant [37].

According to Carvalho and Gomes [38], there are two families of LTPs: LTP1, which has a molecular mass close to 10 kDa, a basic pH and an isoelectric point between 9 and 10, and LTP2, which has a molecular mass of approximately 7 kDa and a high isoelectric point. Both families of LTPs have *in vitro* inhibition activity against fungi and bacteria, which may have great value in programs for the improvement of *Capsicum* resistance to diseases, since the inhibitory action of LTP can be used *in vivo* to combat several pathogens, including *C. gloeosporioides*, with the goal of releasing an anthracnose-resistant cultivar. Some studies, such as that of Wang et al. [39], have shown antimicrobial activity of the mung bean LTP against other agronomically important pathogens such as *Fusarium solani, F. oxysporum, Pythium aphanidermatum* and *Sclerotium rolfsii*.

**Figure 4:**
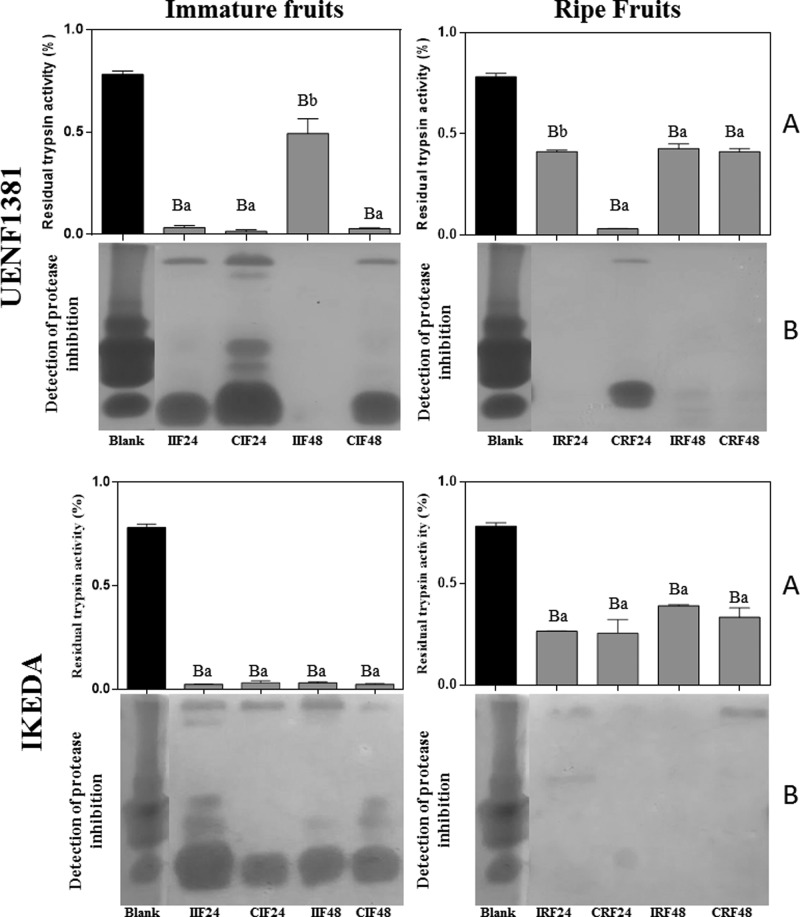
Trypsin inhibition assay Inhibitory activity assay of trypsin (**A**) and reverse zymographic detection of protease inhibition (**B**) on immature and ripe fruits samples of *Capsicum annuum*, from UENF1381 and IKEDA, 24 and 48 h after inoculation with the fungus *Colletotrichum gloeosporioides*. I: inoculated; C: control (not inoculated); Blank **(A)**: commercial trypsin; Blank **(B)**: commercial trypsin inhibitor. Means followed by the same capital letter does not differ in relation to the control of the test, by Tukey’s test at 5% probability. Means followed by the same lowercase letter do not differ from the uninoculated control by the Tukey’s test at 5% probability.

From the trypsin inhibition assay, it was possible to verify that there is inhibition of trypsin in all tested treatments (Figure 4A), with significant differences in all the treatments in respect to the blank. However, only the samples IIF48 and IRF24 from UENF1381 presented significant differences in respect to their individual controls, CIF48 and CRF24, respectively. It was observed for all treatments of ripe fruits, except for CRF24 and IRF24 de UENF1381, that the percentage of inhibition decreased in relation to immature fruits, without significant differences by Tukey’s test. These data are in agreement with the mass spectrometry sequencing, where there was the identification of trypsin inhibition in the immature fruits of IKEDA (Table 3). Pearce et al. [40] showed that some protease inhibitors of wild tomato species were present in a large quantity in immature fruits, acting as defensive products against herbivory, and during maturation of the fruits the levels of inhibitors decreased turning them edible and facilitating seed dispersal. Protease inhibitors were also detected by zymography (Figure 4B), and the results corroborate those from the trypsin inhibition assays (Figure 4A). Ribeiro et al. [20] identified and purified a protein with trypsin inhibitory activity, called *CaTI*, from the *C. annuum* seed. This protein had the ability to permeabilize the plasma membrane of *Saccharomyces cerevisia*e and *Candida albicans* cells after 24 h of growth. Silva et al. [7] reported growth inhibition of the fungi *C. gloeosporioides* and *C. lindemuthianum* by the *CaTI* inhibitor, in addition to showing the ability of the protein to permeabilize the membrane of all the tested fungi, what indicates the importance of protease inhibitors in the defense of plants against several pathogens. The protease inhibitors are constitutively present in all plant kingdoms, being mainly located in the reserve tissues, in addition to being produced in response to injuries caused by insects or even by fungi and bacteria [41–43]. Alternatives aiming at the reduction of pest and disease damages to current crops are of great interest as a whole, mainly for plant breeding, and the use of PR proteins has great potential for such purposes.

**Figure 5:**
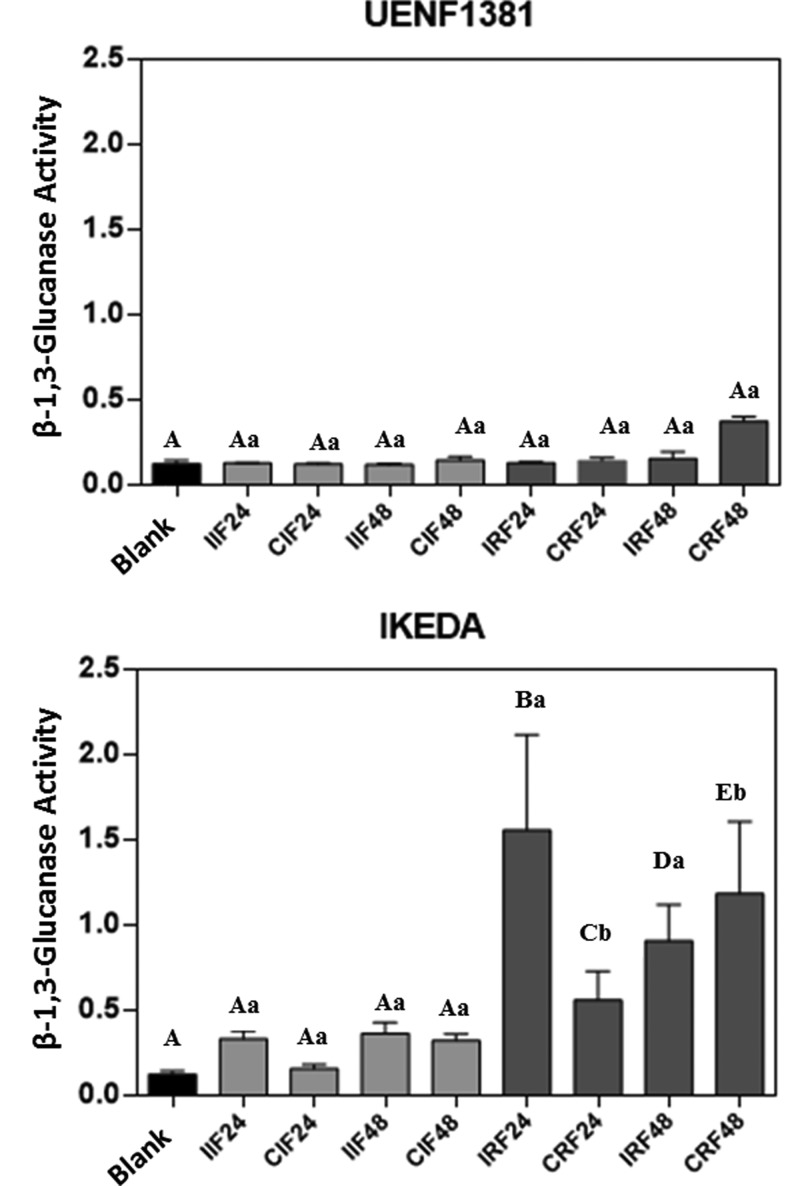
Determination of β-1,3-glucanase activity β-1,3-Glucanase activity of immature and ripe fruits of UENF1381 and IKEDA *Capsicum annuum*, 24 and 48 h after inoculation with the fungus *Colletotrichum gloeosporioides*. I: inoculated; C: control (not inoculated); Blank: assay buffer. Means followed by the same capital letter do not differ in relation to the control of the test by Tukey’s test at 5% probability. Means followed by the same lowercase letter do not differ from the uninoculated control by the Tukey’s test at 5% probability.

The activity of β-1,3-glucanase was detected in all analyzed treatments (24 and 48 h, immature and ripe, UENF1381 and Ikeda) (Figure 5), but with higher activity in ripe fruits of the Ikeda. In the fruits of UENF1381, β-glucanase activity was low and similar at all-time intervals and did not differ significantly between immature and ripe fruits or between treated immature or ripe fruits and their respective controls. In Ikeda fruits, the β-glucanase activity was higher, being predominant in ripe fruits, showing activity well above the control, with significant differences at 0.05% probability for treatments IRF24, IRF48 and CRF48 between each other and in relation to the general control. It should be noted that the β-1,3-glucanase activity in the Ikeda ripe fruits inoculated for 24 h (IRF24) was much higher than in the fruits subjected to the other treatments, differing significantly from all the other treatments analyzed, including the uninoculated control (CRF24). In ripe 48 h inoculated fruits of the Ikeda (IRF48), the difference with respect to the control (CRF48) decreases, but the value remains significantly different from all tested treatments. According to Balasubramanian et al. [44], β-1,3-glucanases play an important role in plant defense by means of hydrolytic degradation of β-1,3/1,6-glucans present in the cell wall of pathogenic fungi, and are used as elicitors that activate a signaling cascade, resulting in the production of other proteins used in plant defense.

Aggarwal et al. [45] quantified the levels of glucanase expression over time in leaves of 12 wheat genotypes inoculated with *Bipolaris sorokiniana* and found that some genotypes were resistant to the fungus; seven genes were identified in the response to the fungus, among them the *Glucanase I* and *Glucanase II* genes. The *Glucanase II* gene was significantly upregulated in resistant genotypes 16 and 24 h after inoculation; its expression levels were increased up to 7-fold compared with the control. It was also observed that the *Glucanase I* and *Glucanase II* genes were not found in the susceptible treatment. These results are consistent with the results obtained in the present work since the highest levels of β-1,3-glucanase activity occurred in ripe fruits of the Ikeda. However, the Ikeda in this experiment are considered susceptible to treatment.

## Conclusions

In conclusion, it is shown that *C. annuum* fruits present several low molecular weight proteins in the diverse analyzed treatments, particularly antimicrobial peptides such as defensin, LTP and protease inhibitor. These proteins inhibit the early growth of *C. gloeosporioides*, causing hyphal morphological alterations. The findings reported in the present study on the presence of different defense proteins and antimicrobial peptides in distinct genotypes of *Capsicum*, which are differentially expressed in ripe and immature fruits, suggest an important role for these proteins, especially in constitutive host defense mechanisms against microbial pathogens. This may contribute for the development of biological control of fungal pathogens typical of *Capsicum.*

## Supporting information

**supplementary Figure 1:** Supplementary material (A) Humid chamber with immature fruits after injury created in the middle region of each fruit with a sterilized needle followed by inoculation with 20 μL of the *C. gloeosporioides* fungus spore solution. (B) Immature and ripe fruits ranging from 2 to 5 cm in length and 1 to 1.5 g per fruit after 48h of inoculation with *C. gloeosporioides* fungus spore. Arrows indicate the point of inoculation.

## Abbreviations

AMP: antimicrobial peptide
LTP: lipid transfer protein
PDA: potato dextrose agar
