# Supplementary material for: Biochemical analysis of antimicrobial peptides in two different Capsicum genotypes after fruit infection by Colletotrichum gloeosporioides
Source: Biosci Rep. 2019 Apr 23;39(4):BSR20181889. doi: 10.1042/BSR20181889 (PMC6481241; doi:10.1042/BSR20181889)
Supplement: Supplementary file 1 [file bsr20181889_Supp1.pdf]

A

Capsicum annuum

UENF 1381

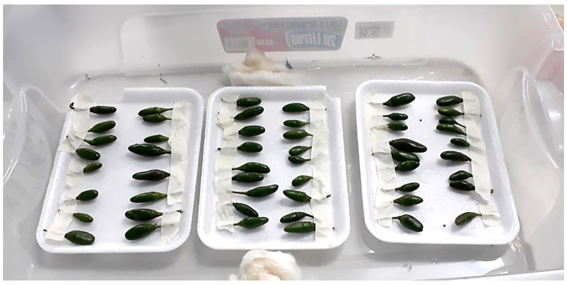

B

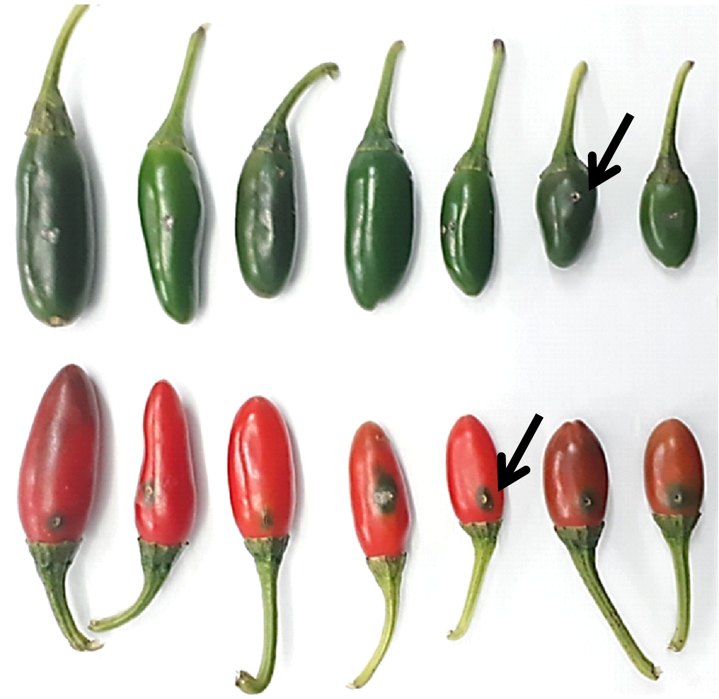

Supplementary material. (A) Humid chamber with immature fruits after injury created in the middle region of each fruit with a sterilized needle followed by inoculation with 20 µL of the *C. gloeosporioides* fungus spore solution. (B) Immature and ripe fruits ranging from 2 to 5 cm in length and 1 to 1.5 g per fruit after 48h of inoculation with *C. gloeosporioides* fungus spore. Arrows indicate the point of inoculation.
